# Susceptibility of Multidrug-Resistant and Biofilm-Forming Uropathogens to Mexican Oregano Essential Oil

**DOI:** 10.3390/antibiotics8040186

**Published:** 2019-10-15

**Authors:** Karen Arely Zapién-Chavarría, Alejandro Plascencia-Terrazas, María Georgina Venegas-Ortega, Mauricio Varillas-Torres, Blanca Estela Rivera-Chavira, Jaime Raúl Adame-Gallegos, María Olga González-Rangel, Guadalupe Virginia Nevárez-Moorillón

**Affiliations:** 1 Facultad de Ciencias Químicas, Universidad Autónoma de Chihuahua, Circuito Universitario s/n Campus Universitario II, 31125 Chihuahua, Chihuahua, Mexico; 2 Research Group of Bioprocesses and Bioproducts, Department of Food Research, School of Chemistry, Universidad Autónoma de Coahuila, 25280 Saltillo, Coahuila, Mexico

**Keywords:** multidrug resistance, Mexican oregano, Biofilm formation, phylogenetic analysis, urinary tract infections

## Abstract

Antibiotic resistance along with biofilm formation increases the difficulty for antibiotic therapy in urinary tract infections. Bioactive molecules derived from plants, such as those present in essential oils, can be used to treat bacterial infections. Oregano is one of the spices to have antimicrobial activity. Therefore, three Mexican oregano essential oils (two *Lippia berlandieri* Schauer and one *Poliomintha longiflora*) were tested for antimicrobial capacity against multidrug-resistant, biofilm-forming bacterial isolates. Clinical isolates from urinary tract infections were tested for antibiotic resistance. Multidrug-resistant isolates were evaluated for biofilm formation, and Mexican oregano antimicrobial effect was determined by the minimal inhibitory (CMI) and minimal bactericidal concentrations (CMB). The selected isolates were identified by molecular phylogenetic analysis. Sixty-one isolates were included in the study; twenty were characterized as multidrug-resistant and from those, six were strong biofilm formers. Three isolates were identified as *Escherichia coli*, two as *Pseudomonas aeruginosa* and one as *Enterococcus faecalis* based on the phylogenetic analysis of 16 S rRNA gene sequences. The antimicrobial effect was bactericidal; *E. faecalis* was the most susceptible (<200 mg/L CMI/CMB), and *P. aeruginosa* was the most resistant (>2,000 mg/L CMI/CMB). There was a range of 500-1000 mg/L (CMI/CMB) for the *E. coli* isolates. Mexican oregano essential oils demonstrated antimicrobial efficacy against multidrug-resistant clinical isolates.

## 1. Introduction

Urinary tract infections (UTI) are among the most persistent clinical infections, in part because the location of the infectious site hampers the efficacy of antibiotics. These types of infectious processes are more common in women because of their anatomic structures, and in many cases, are resistant to antibiotic therapy [1]. Cases of UTI infections in hospitalized patients are also related to the use of urinary catheters, particularly in elderly patients that usually present complicated infections [2]. 

On the other hand, biofilm formation in clinical infections have been reported in as much as 80% of cases, and 65% of hospitalized-related infections and UTI are commonly associated with biofilm-forming pathogens [3]. It has been proposed that the capacity of adherence by pathogenic strains is a critical factor in bladder colonization, and that biofilm formation is a mechanism of survival to stressful conditions [4]. The correlation between virulence genes and biofilm formation in uropathogenic isolates has been reported in several studies, but the results are not conclusive [5,6,7,8]. In UTIs related to catheters in hospitalized patients, up to 73% of the bacterial isolates were biofilm-forming and were more resistant to antibiotics than those not able to form biofilm [9]. 

In many cases, UTI-related bacterial isolates are resistant to commonly used antibiotics, making the selection of the therapeutic scheme even more complicated. Treatment is even more difficult if it is considered that many isolates are multidrug-resistant [7,10]. The necessity for new antibiotics and new treatments for clinical infections has led to the study of aromatic plants that contain bioactive molecules as their constituents [11]. Oregano essential oil includes the isomers thymol and carvacrol that are responsible for the antimicrobial activity [12,13]. Several plants are identified as oregano, including *Lippia berlandieri* Schauer (Mexican oregano) and *Poliomintha longiflora* (Mexican mountain oregano). The two species have differences on the percentages of thymol and carvacrol contained in their essential oils [12,13]. 

The aim of this work was to determine the antimicrobial resistance of bacterial isolates from the urine of UTI patients and to evaluate biofilm formation of the multidrug-resistant isolates. Finally, the antimicrobial effect of three essential oils of Mexican oregano was tested against the biofilm-forming, multidrug-resistant isolates.

## 2. Results

### 2.1. Bacterial Isolates from UTI Patients

Bacterial isolates were collected at the University Hospital from urine samples of UTI patients by the hospital personnel and were provided as pure culture, along with the information of patient´s age, gender and if they were hospitalized or outpatients. A total of 61 isolates were collected and included in this study; regarding the age of the patients, the range was between 13 and 92 years old, with an average of 53 years. Patients were 73% female (45/60) and 27% male (16/60). In the study, 57% of the patients were external (35/60), and 43% (26/60) of them were hospitalized at the moment of the study. Bacterial isolates were identified as I + number for inpatients and E + number for external patients. 

**Figure 1:**
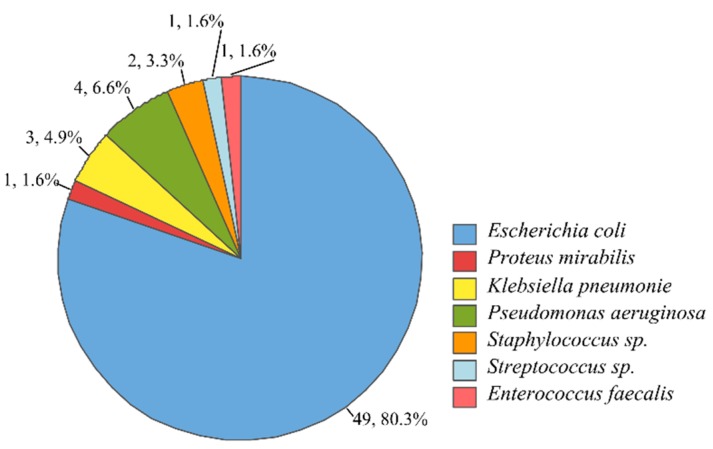
Bacterial isolates from urinary tract infection (UTI) patients and presumptive identification based on biochemical tests. For each group, the number of isolates and the respective percentage is included in the figure as a label.

Based on the results of biochemical tests, seven microbial groups were identified, including *Escherichia coli*, *Proteus mirabilis*, *Klebsiella pneumoniae*, *Pseudomonas aeruginosa*, coagulase-negative Staphylococcus spp., *Streptococcus* spp., and *Enterococcus faecalis*. From all isolates, 92% were Gram-negative, and 8% were Gram-positive isolates. The percentage of isolates of each group is presented in Figure 1.

### 2.2. Antibiotic Resistance of Bacterial Isolates from the Urine of UTI Patients

**Figure 2:**
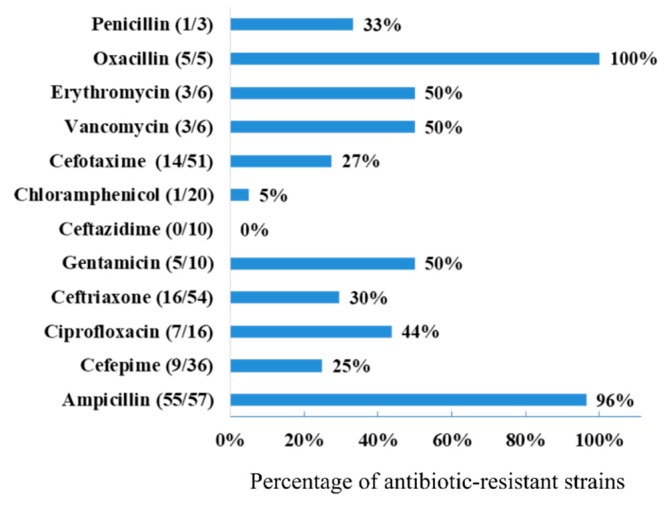
Percentage of antibiotic resistance of bacterial isolates from UTI patients.

Antibiotic resistance was tested with five antibiotics, selected from the ones that are commonly used in UTI treatment for each pathogen. Figure 2 shows the percentage of resistant isolates to the antibiotics tested. Isolates were more resistant to Ampicillin (96%) and Oxacillin (for Gram-positive). All isolates tested for Ceftazidime were susceptible. 

Based on the criteria proposed for identification of multidrug-resistant bacteria [14], 20 bacterial isolates (33%) were identified as resistant to at least three antibiotics and further selected for this study. Fifteen of the 20 isolates were identified as *E. coli*, two belong to the coagulase-negative *Staphylococcus* sp. group, two were identified as *P. aeruginosa*, and one was identified as *E. faecalis*. Half of the *Staphylococcus* sp. and *P. aeruginosa* isolates were multi resistant, while 32.6% of the *E. coli* isolates were resistant to at least three antibiotics. The information on the antibiotic-resistance of each multidrug-resistant isolates is included in Table 1. Nine of the 20 isolates were from hospitalized patients, while 11 were obtained from UTI outpatient samples.

### 2.3. Biofilm Formation of Multidrug-Resistant Bacterial Isolates

Biofilm formation was tested in the multidrug-resistant isolated by two methods, a qualitative test in glass tubes, and the quantitative microplate test. In the first test, the biofilm formed by the isolates in liquid growth adhered to the glass of the test tube was carefully removed. The biofilm was then stained with safranin, and the colored EPS was visually classified as negative, or with various degrees of positive (+, ++, +++). Results of the test are included in Table 1. 

The results of the quantitative determination of adhesion to microplate proposed by Stepanovic [15] are also included in Table 1. Results demonstrated that five isolates were negative for both tests, while six were identified as with weak adherence in the test tube, and non-adherent in the microplate test. Two isolates were highly positive (+++) in the test tube and moderately adherent with the microplate test. Differences in the results of both tests can be attributed to differential adherence capacity to the two substrates. Overall, six isolates were identified as strongly adherent in the microplate test, and those were selected to test the antimicrobial capacity of Mexican oregano essential oils.

### 2.4. The Antimicrobial Capacity of Mexican Oregano Essential Oil Against Selected Isolates

**Figure 3:**
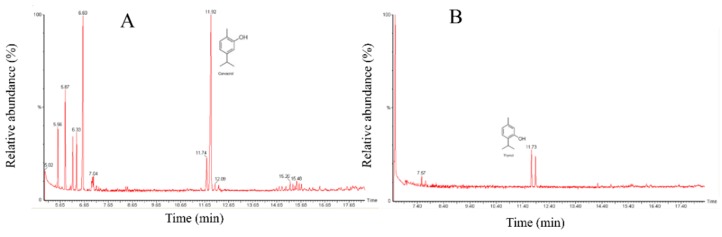

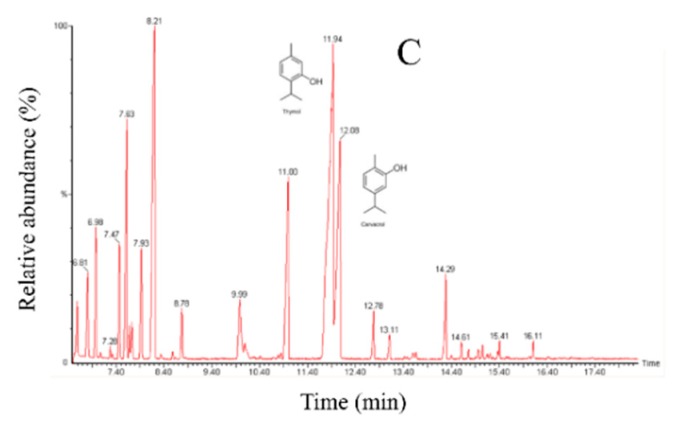
Gas-Chromatography chromatogram of Mexican oregano essential oils. (**A**)*. Lippia berlandieri* Schauer with a higher proportion of carvacrol (EO1). (**B**). *Lippia berlandieri* Schauer with higher proportion of thymol (EO2). (**C**). *Poliomintha longiflora*, with a higher proportion of thymol.

Six isolates were selected based on their profile of multidrug resistance and biofilm formation to determine their susceptibility to Mexican oregano essential oils. Three different essential oils were tested. There are many different plant species identified as oregano, but they share the presence of thymol and carvacrol as main components. In Mexico, one of the most common oregano species is *Lippia berlandieri* (Schauer) (also as *Lippia greveolens* Kunth), which has a stronger flavor than European oregano (*Origanum vulgare*). Another oregano spice used in Mexico, identified as mountain oregano (*Poliomintha longiflora*), that is found in the Northeast part of Mexico, was also included in this study [16]. Two chemotypes of *L. berlandieri* were studied, one with a higher concentration of carvacrol with a relative proportion of 60/20 carvacrol/thymol (EO1), and the other with a higher proportion of thymol relative proportion 40/20 (EO2). The characterization of the *P. longiflora* EO showed a higher proportion of thymol than carvacrol (Figure 3).

The antimicrobial effect of the Mexican oregano EO was tested against the multidrug-resistant selected isolates, by the determination of the minimal inhibitory and minimal bactericidal concentration, as presented in Table 2. The isolates selected included three *E. coli*, two *P. aeruginosa*, and the *E. faecalis* isolate. The most susceptible bacterial isolate was *E. faecalis*, which had a CMI/CMB lower than 200 mg/L for the three EOs, that was the lowest concentration tested. As expected, *P. aeruginosa* isolates were resistant even when the concentration of oregano EO was of 2000 mg/L. Regarding the *E. coli* isolates; there was a differential effect, with CMI/CMB values of 500–1000 mg/L. The effect of *L. berlandieri* EO was similar, regardless of their carvacrol/thymol proportion, and the effect was different from *P. longiflora* in two of the *E. coli* isolates. In all the cases, the effect was bactericidal rather than bacteriostastic.

### 2.5. Molecular Identification of Multidrug-Resistant, Biofilm-Forming Isolates from the Urine of UTI Patients

Molecular characterization of the isolates tested for the antimicrobial effect of Mexican oregano essential oil was done to characterize those that were multidrug-resistant and showed strong adherence in the microplate test for biofilm formation. The phylogenetic analysis included the forward and reverse sequences obtained from the PCR product. Initially, the identification was made using the BLAST tool (NCBI). Sequence identities were considered admissible with similarity percentages of >99.5% with the sequences found in the database. Phylogenetic trees were developed using the maximum likelihood approach, with the Kimura2 model and a Gamma distribution (K2 + G). Precision was tested by the Bootstrap analysis with 1000 repetitions. 

**Figure 4:**
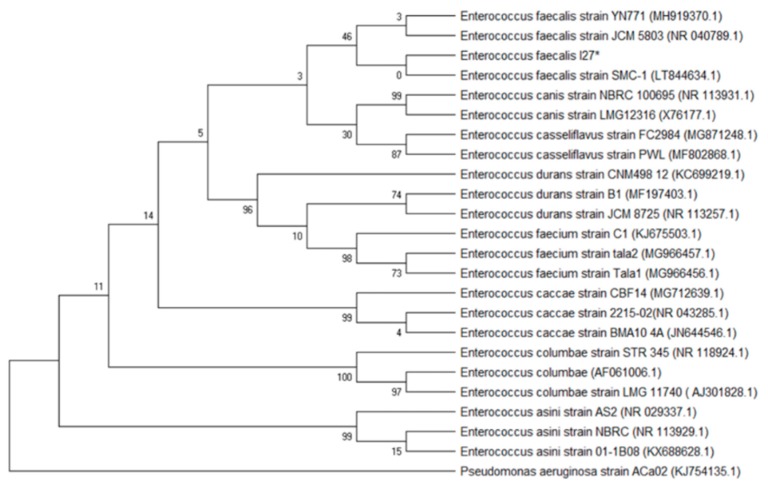
Phylogenetic tree based on the 16 S rRNA gene sequences of *Enteroccus* (I27) and reference sequences. The analysis included 24 sequences selected from GenBank, and the access number is given in parenthesis. An asterisk identifies the bacterial isolate from this study.

**Figure 5:**
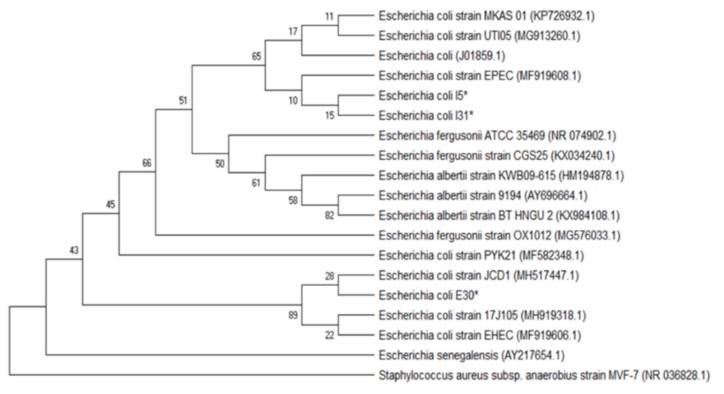
Phylogenetic tree based on the 16 S rRNA gene sequences of the *Eschericha coli* isolates and reference sequences. The analysis included 19 sequences selected from GenBank, and the access number is given in parenthesis. An asterisk identifies the bacterial isolates from this study.

**Figure 6:**
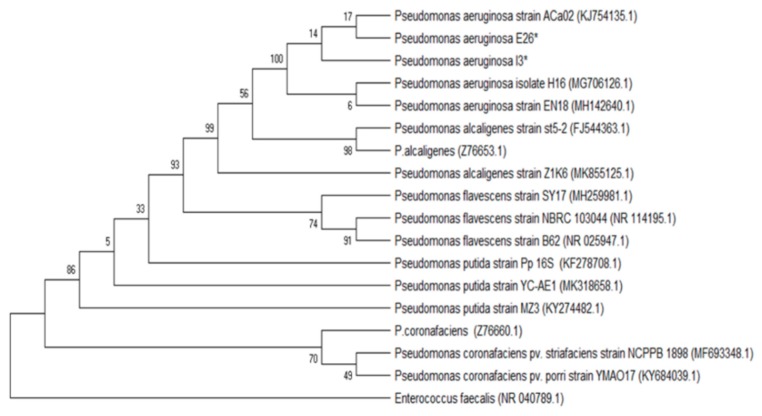
Phylogenetic tree based on the 16 S rRNA gene sequences of the *Pseudomonas aeruginosa* isolates and reference sequences. The analysis included 18 sequences selected from GenBank, and the access number is given in parenthesis. An asterisk identifies the bacterial isolates from this study.

Figure 4 shows the phylogenetic tree for the *Enterococcus* I27 isolate (GeneBank access number MN305687), identified as *E. faecalis*. Figure 5 includes the *E. coli* isolates I5 (GeneBank access number MN305691), E30 (GeneBank access number MN305690) and I31 (GeneBank access number MN305692). In the phylogenetic tree, isolates I5 and I31 were clustered close to each other and separated from E30. Figure 6 includes the isolates E26 (GeneBank access number MN305688) and I3 (GeneBank access number MN305689) that were identified as *P. aeruginosa*.

## 3. Discussion

The increase of multidrug-resistant isolates in clinical isolates is one of the most significant challenges of public human health now. The appearance of resistant isolates to new antibiotics takes less time than is required to develop new antimicrobial substances. The abuse of antibiotics, not only for therapeutics but also in agriculture and animal production, has led to the wide dispersion of antibiotic-resistant genes [17]. 

In this report, resistance to β-lactam antibiotics is elevated (penicillin 33.3%, oxacillin 100%, and ampicillin 96%), which is expected since it is one of the antibiotics most frequently used in therapeutics [18]. On the other hand, low-resistant percentages (25–30%) were presented to third and fourth generation cephalosporins and isolates were 100% susceptibility to ceftazidime. Dumaru et al. [7] reported high resistance to ceftazidime from *E. coli* clinical isolates in Nepal, where this antibiotic is used to treat UTI cases. In a retrospective 5-years analysis of antibiotic resistance in UTI-related *E. coli* isolates from Australia, an increase was observed in antibiotic resistance to ampicillin, trimethoprim and cefazolin [10]. In a report from Aguascalientes, Mexico, UTI-related *E. coli* isolates were highly resistant to ampicillin and trimethoprim-sulfamethoxazole (more than 70% of the isolates) [6]. The surveillance of antibiotic resistance is not only crucial for the treatment in clinical infections but also provide information on the presence of antibiotic resistance genes in a particular geographical area.

The environment can be a source of antibiotic resistance genes, but in the clinical environment the presence of multi-resistant bacteria can be stimulated [17]. Also, clinical conditions such as urinary catheters can increase the frequency of UTI with multidrug-resistant bacterial isolates [4]. However, the prevalence of multidrug-resistant isolates in UTI infections of outbound patients should not be underestimated [6]. In this report, more than half of the multidrug-resistant isolates were from UTI outpatients (11/20); therefore, the surveillance of antibiotic resistance patterns need to be carefully monitored not only in hospitalized patients but in community-acquired infections. When food consumer habits were included in the evaluation of risks associated to community-acquired UTI infections by *E. coli* isolates, low antibiotic resistance of the isolates was related to consumption of fruits such as nectarine and apples, peppers or fresh herbs and even cooked beef. No explanation for this protective phenomenon was provided by the authors, which also explored other risks factors such as travel or contact with pets [19].

Regarding the genera of multidrug-resistant bacterial isolates obtained from UTI patients in this study, a high percentage was related to bacteria usually found in the gastrointestinal tract (*E. coli* and *E. faecalis*), that account for 80% of the isolates. *P. aeruginosa* isolates were also present in the group (2/20) that also included coagulase-negative *Staphylococcus* sp. These microbial species are reported as the most commonly related UTI genera [4,9]. 

On the other hand, bacterial adhesion capacity has been related to the presence of specific pilis or other virulence genes. In chronic infections such as wounds, otitis, endocarditis, and recurrent UTI, the pathogenic bacterial isolates have been identified as biofilm producers [3]. In a report from Nepal, 63% of Gram-negative antibiotic-resistant clinical isolates were positive for biofilm formation [7]. Biofilm formation capacity can be considered a high-risk pathogenic treat, especially in UTI processes since cells within biofilm are less susceptible to antibiotics. Hospitalized patients with catheters are at higher risks of developing UTI infections with biofilm-forming and highly virulent isolates. In this report, adherence capacity was tested in multidrug-resistant isolates, and 30% were highly adherent based on the microplate adherence test, although other isolates were also moderate or weakly adherent.

The prevalence of *Enterococcus* spp. in UTI has increased in recent years, as well as the multidrug resistance on the isolates [8]. The resistance to glycopeptides such as vancomycin and β-lactam has been reported [4]. On the other hand, *Enteroccus* spp. have a strong capacity to form biofilms, and this capacity is correlated with other virulence factors such as collagen adhesion, surface proteins, and biofilm-associated-pilis [4,20]. Although in our study there is only one isolate of *E. faecalis* identified as biofilm-forming and multidrug-resistant, it is important to monitor the presence of this uropathogenic bacterium, as well as their antibiotic resistance patterns.

Although antibiotic therapy is highly effective and can be considered as one of the significant contributions to improving human health, the use of traditional medicinal knowledge cannot be ignored. In developing countries, up to 80% of the population uses herbal-related medicine as their primary healthcare strategy, including infusions of medicinal plants and the use of essential oils as unguents [21]. Even with other natural products such as honey, there are reports that suggest the use of these compounds against antibiotic-resistant isolates isolated from the urinary tract of pregnant women [1].

The identification of bioactive compounds present in plants can provide novel molecules for antibiotic development. In the case of essential oils however, although major components have antimicrobial effect, the combination of all compounds present increases their biological activity [11]. One of the essential oils with higher antimicrobial capacity is Oregano, and its activity has been related to thymol and carvacrol. Both thymol and carvacrol are synthesized from p-cymene, so that proportions of the terpenes can vary depending on the plant species [16]. There are more than 60 plants identified as oregano worldwide that share the presence of thymol and carvacrol in their essential oil. In Mexico, *Lippia berlandieri* Schauer (synonym of *Lippia graveolens*) is the most commonly found oregano plant, and it usually presents a higher concentration of carvacrol than its European counterpart, *Origanum vulgare*. Since carvacrol has a stronger odor and stringer flavor, the study of different chemotypes of *L. berlandieri* could identify plants that can have a higher commercial value [16]. On the other hand, *Poliomintha longiflora* is known as mountain oregano, and it presents a chemical composition more related to European varieties [13]. 

The antimicrobial effect of the essential oil of three different essential oils from Mexican oreganos was tested against the biofilm-forming, multidrug-resistant isolates selected in this study. As expected, *P. aeruginosa* was resistant to the essential oils (CMI/CMB higher than 2000 mg/L), but *E. coli* isolates were susceptible in the 500–1000 mg/L range, and the effect was bactericidal. It is essential to note the effect of the three essential oils against the *E. faecalis* isolate, with CMI/CMB lower than the lowest concentration tested (250 mg/L). The results of antimicrobial efficacy of Mexican oregano are similar to previous reports [13]. The different concentrations of carvacrol and thymol in the *L. berlandieri* essential oils (EO1 and EO2) did not produce a difference in the antimicrobial activity presented. Both carvacrol and thymol can produce rupture of the bacterial cell membrane, due to their hydrophobic nature [22,23]. Gram-negative bacteria are more resistant to terpenes than Gram-positive bacteria due to their external membrane [13].

In order to assure that the isolates tested were correctly characterized, the molecular identification of the six isolates tested was made by 16 S rRNA gene sequencing, and their phylogenic aggrupation is presented in Figure 4, Figure 5 and Figure 6. Although molecular characterization of all clinical isolates might not be feasible yet in clinical laboratories, it is essential to fully characterize isolates with properties such as the ones selected, that are multidrug-resistant and have the capacity to form biofilms [24]. 

There are many aspects that need to be considered for the use of essential oils or their components in the treatment of UTI patients, including the delivery of the compounds to the infection site, as well as their degradation pathways in the human body. The observance of antimicrobial efficacy of Mexican oregano against multidrug-resistant bacterial isolates can be useful in the identification of chemical structures that can be used as backbones on the preparation of new antimicrobial substances. 

## 4. Materials and Methods

### 4.1. Bacterial Isolates

The Diagnostic Laboratory at the University Hospital (Universidad Autónoma de Chihuahua, México) provided the bacterial isolates (collected during May–July 2016). Bacterial cultures were isolated from urine samples of patients with urinary tract infections (UTI) that were submitted to the microbiology lab for analysis. Samples were from inpatients wards and outpatients. Bacterial isolates were identified based on colonial and cellular morphology, as well as biochemical tests [25]. For the multidrug-resistant isolates, bacterial identification was confirmed using API 20E Test System (bioMérieux, Marcy-l'Étoile, France).

### 4.2. Antibiotic Susceptibility Test 

Bacterial stains antibiotic susceptibility was tested using Muller-Hinton agar by the Kirby Bauer disc diffusion method [26]. The antibiotics used included Ampicillin (10 µg), Cefepime (30 µg), Ciprofloxacin (5 µg) Ceftriaxone (30 µg), Gentamicin (10 µg), Ceftazidime (30 µg), Chloramphenicol (30 µg), Cefotaxime (30 µg), Vancomycin (30 µg), Erythromycin (15 µg), Oxacillin (1 µg) and Penicillin (10 µg). Susceptibility and resistance were determined according to the manufacturer instructions (BD BBL Sensi-Disc Antimicrobial Susceptibility Test Discs. BD, Maryland, USA).

### 4.3. Biofilm Formation Test

Biofilm formation by the clinical bacterial isolates was tested by two methods, the qualitative biofilm formation test in glass test tubes and the microplate test using crystal violet solution. For the qualitative test, the isolates were inoculated in glass test tubes with Tryptic Soy Broth (TSB) (BIOXON, Mexico City, Mexico) and incubated for 24 h at 37 °C. After incubation, the broth was carefully removed, and 2 mL of safranin was added to the tube and rolled over the sides to stain the remaining biofilm. The excess of dye solution was removed, and the tube stored in inverted position for additional 24 h. A positive result was recorded when the stained biofilm was evident in the tube. The microplate test was done according to Stepanovic et al. [15], using TSB as medium for bacterial growth, and 2% crystal violet solution for staining of the formed biofilm. The grade of adherence was determined as describes in the method. All test were done by triplicate.

### 4.4. Oregano Essential Oil Extraction and Analysis

Cirena (Centro de Investigación en Recursos Naturales, Salaices López Chihuahua, Mexico) provided essential oil from *L. berlandieri* Schauer and *P. longiflora* oregano species. Two different *L. berlandieri* essential oils with different proportion of thymol and carvacrol and one essential oil of *P. longiflora* were included in the study. The essential oils were analyzed to determine the relative concentration of the active components carvacrol and thymol [13]. Essential oils were analyzed in a GC Perkin Elmer Turbo Mass Gold MS-Auto system XLTM (Perkin-Elmer, Norwalk, CT, USA) with a splitless injector and 70 eV electronic fragmentation detector, using a TG-5 SiLMS column (30 m × 0.25 I.D. × 0.25 μm) (Thermo Fisher Scientific, Whaltman MA, USA). Helium was used as the carrier gas, at 1 mL/min flux, and the following conditions were used for analysis: the temperature of the injector was 220 °C; the initial oven temperature was 50 °C held for 2 min, followed by a ramp-up of 10 °C/min up to 130 °C, a second ramp-up of 5 °C/min up to 150 °C and a third ramp-up of 30 °C/min up to 190 °C, and held at the final temperature for 3 min. The essential oils were prepared in 200 mg/L solutions using hexane as solvent; the volume used for injection was 1 μL. The main components thymol and carvacrol were identified by their retention time and the mass profile of the compounds available from the US National Institute of Standard Technology (NIST) library.

### 4.5. Antimicrobial Activity of Mexican Oregano Essential Oil

For evaluation of the antimicrobial activity of the Mexican oregano essential oils, the clinical bacterial isolates were tested for determination of the Minimal Inhibitory and Minimal Bactericidal concentration, as described before [13] adapted to microplate volumes. Briefly, the Oregano essential oils were dissolved in ethanol (100% pure ethanol) to a 0.5% (w/v) initial solution and further diluted in TSB to achieve a final concentration in the broth of 250, 500, 750, 1000, 1500 and 2000 mg/L. Also, clinical bacterial isolates were grown in TSB overnight, and a concentration equivalent to 0.5 McFarland tube was prepared in saline solution (0.85% NaCl) and added to the microplate wells (50 μL). Three controls were included, a control for bacterial growth with TSB but no essential oil added, a control to test if the concentration of ethanol used for essential oil suspension was not bactericidal, and a negative control, with essential oil and saline solution without bacterial inoculum. The microplate was incubated at 37 °C for 24 h. After incubation, Minimal Inhibitory Concentration (MIC) values were determined as the lowest concentration without observed growth. For determination of the Minimal Bactericidal Concentration (MBC), a loopful from the wells where no evident growth was observed, were inoculated onto Tryptic Soy Agar (TSA) and incubated for 24 h at 37 °C. MBC was determined as the minimal concentration with no growth on the TSA plates. 

### 4.6. Bacterial Isolates Molecular Identification

For molecular identification, the bacterial isolates were analyzed by 16 S rRNA gene sequence. The DNA extraction was done from 2 mL bacterial cultures via the phenol-chloroform extraction protocol [27], total DNA was quantified and stored at −20 °C. Extracted genomic DNA was subjected to DNA amplification by PCR using 27 f (5´-agagtttgatcctggctcag-3´) and 1492 r (5´-acggctaccttgttacgactt-3´) primers [28]. Each reaction was done in a final volume of 50 μL containing 29 μL of sterile Milli Q water, 7 μL of 10× Taq buffer, 4 μL of each primer, 1 μL of dNTP Mix, 1 μL of Taq Polymerase and 4 μL of extracted genomic DNA (100 ng/μL). The conditions for PCR amplification included an initial denaturation step (95 °C, 10 min), 25 amplification cycles of denaturation (93 °C, 1 min each), annealing (50 °C, 1 min each), elongation (72 °C, 1:30 min each) and final extension step (72 °C, 10 min). PCR products were analyzed and purified in agarose gel electrophoresis (1.5%). The PCR product was sequenced by Psomagen (Rockville, MD, USA) by Next Generation Sequencing methodology. 

The sequence information obtained from the clinical isolates were used to construct phylogenetic trees. Initially, the forward, reverse and complimentary reverse sequences were identified using the search algorithms of BLAST (Basic Local Alignment Search Tool) from NCBI (www.ncbi.nih.gov). The sequences were edited using the Seaview software version 9.2. Sequences were compared with 16S rDNA gene sequences selected from GenBank, using BLAST by multiple alignments using CLUSTALX2 version 2.1 and edited in Seaview 9.2. The software used for the phylogenetic analysis was Mega-X version 10.0.5, using 1000 repetitions in the Bootstrap analysis [29,30]. Assembled sequences were submitted to GenBank, and access numbers MN305687 to MN305692 were assigned to the clinical isolates reported in this study. 

## 5. Conclusions

From 61 isolates from urine samples of UTI patients, 20 were multidrug-resistant and were tested for biofilm formation. Six isolates were further selected and were identified by biochemical tests and molecular 16 S rRNA gene sequencing as *E. coli* (3), *P. aeruginosa* (2) and *E. faecalis*.

Three Mexican oregano essential oils, two *L. berlandieri*, and one *P. longiflora* were tested for their antimicrobial capacity against the multidrug-resistant, biofilm-formers isolates. The effect observed was bactericidal, and *E. faecalis* was the most sensitive isolate, followed by the *E. coli* isolates. *P. aeruginosa* was resistant to more than 2000 mg/L. All essential oils showed similar effect, except for the *P. longiflora* essential oil against two of the *E. coli* isolates.

Although there are many questions still to be solved, such as delivery at the infection site or resistance to the bioactive molecules in essential oils, they can be considered for complementary treatment of urinary tract infections.

## Figures and Tables

**Table 1:** Multidrug-resistant clinical isolates from UTI patients and biofilm formation based on the glass tube qualitative test and the quantitative test in a microplate.

Isolates ID	Isolates Identification	Antibiotic Resistance ^a^	Biofilm Formation Test in Glass Tubes ^b^	Biofilm Formation Microplate Test ^c^
E2	*Staphylococcus sp. coagulase negative*	VA, E, OX, P	−	Non-adherent
I3	*Pseudomonas aeruginosa*	AM, CIP, CRO, CL	++	Strongly adherent
I5	*E. coli*	AM, CIP, GM	+	Strongly adherent
I7	*E. coli*	AM, CIP, CRO, GM	+	Non-adherent
E9	*E. coli*	AM, CRO, GM	−	Non-adherent
E10	*E. coli*	AM, CRO, CTX	−	Non-adherent
I13	*E. coli*	AM, CRO, GM, CTX	+	Non-adherent
E21	*E. coli*	AM, CIP, CRO, CTX	−	Non-adherent
I24	*Staphylococcus sp. coagulase negative*	VA, E, OX, P	+++	Moderately adherent
E26	*Pseudomonas aeruginosa*	AM, CRO, CTX, OX	+++	Strongly adherent
I27	*Enterococcus faecalis*	VA, E, OX	+	Strongly adherent
E30	*E. coli*	AM, FEP, CRO, CTX	+	Strongly adherent
I31	*E. coli*	AM, FEP, CRO, CTX	++	Strongly adherent
E36	*E. coli*	AM, FEP, CRO, CTX	+++	Moderately adherent
I38	*E. coli*	AM, FEP, CRO, CTX	+	Non-adherent
E40	*E. coli*	AM, FEP, CRO, CTX	−	Weakly adherent
E50	*E. coli*	AM, FEP, CRO, CTX	+	Non-adherent
E51	*E. coli*	AM, FEP, CRO, CTX	+	Non-adherent
I52	*E. coli*	AM, FEP, CRO, CTX	−	Non-adherent
E53	*E. coli*	AM, FEP, CRO, CTX	+	Non-adherent

^a^ Antibiotic resistance: AM = Ampicillin 10 µg. FEP = Cefepime 30 µg. CIP = Ciprofloxacin 5 µg. CRO=Ceftriaxone 30 µg. GM = Gentamicin 10 µg. CL = Chloramphenicol 30 µg. CTX = Cefotaxime 30 µg. VA=Vancomycin 30 µg. E = Erythromycin 15 µg. OX = Oxacillin 1 µg. P = Penicillin 10 µg. ^b^ Qualitative evaluation of biofilm formation in glass tubes. The signs are relative abundance of biofilm colored with safranin. ^c^ Biofilm formation according to Stepanovic et al. [15] Negative control O.D. 0.161.

**Table 2:** Minimal inhibitory concentration and minimal bactericidal concentration of three different Mexican oregano essential oils against biofilm-forming multidrug-resistant bacterial isolates from UTI urine samples.

Isolates ID	*Lippia berlandieri* EO1		*Lippia berlandieri* EO2		*Poliomintha longiflora*	
	MIC (mg/L)	MBC (mg/L)	MIC (mg/L)	MBC (mg/L)	MIC (mg/L)	MBC (mg/L)
I3 *Pseudomonas aeruginosa*	>2000	>2000	>2000	>2000	>2000	>2000
I5 *E. coli*	500	500	500	500	500	500
E26 *Pseudomonas aeruginosa*	>2000	>2000	>2000	>2000	>2000	>2000
I27 *Enterococcus faecalis*	<250	<250	<250	<250	<250	<250
E30 *E. coli*	750	750	750	750	1000	1000
I31 *E. coli*	500	500	500	500	750	750
I3 *Pseudomonas aeruginosa*	>2000	>2000	>2000	>2000	>2000	>2000
